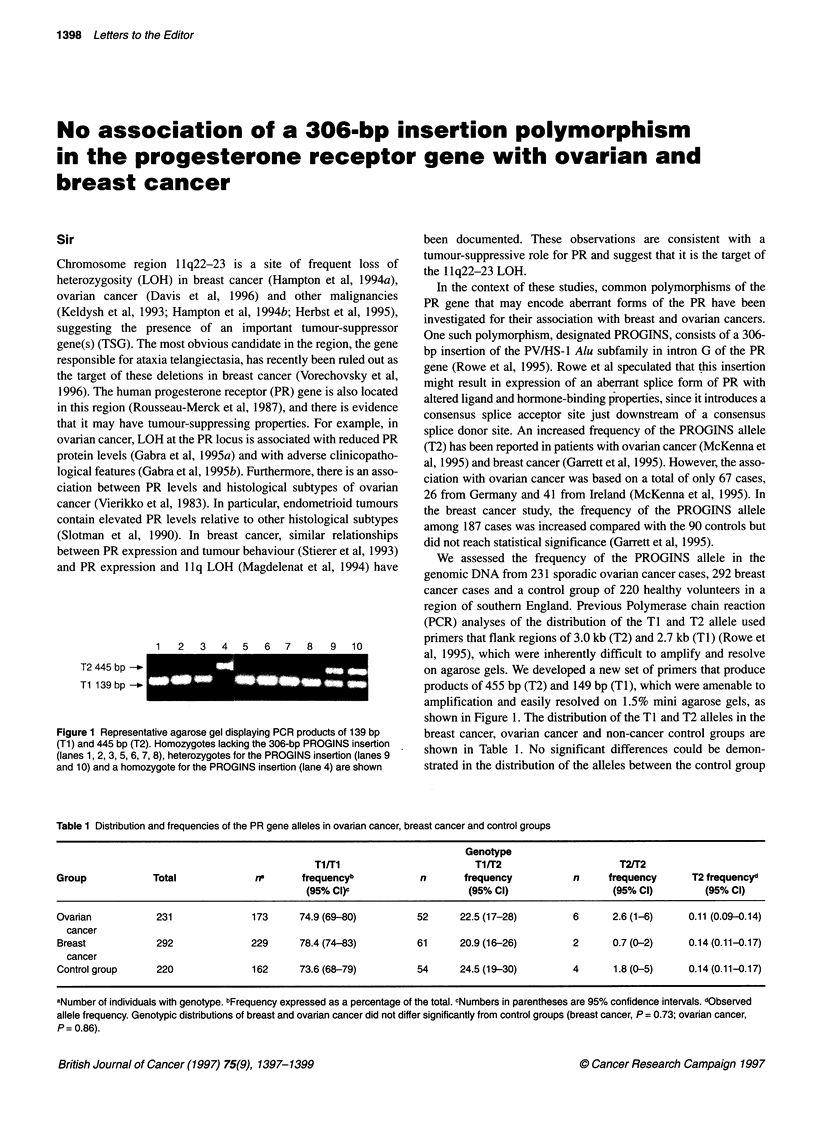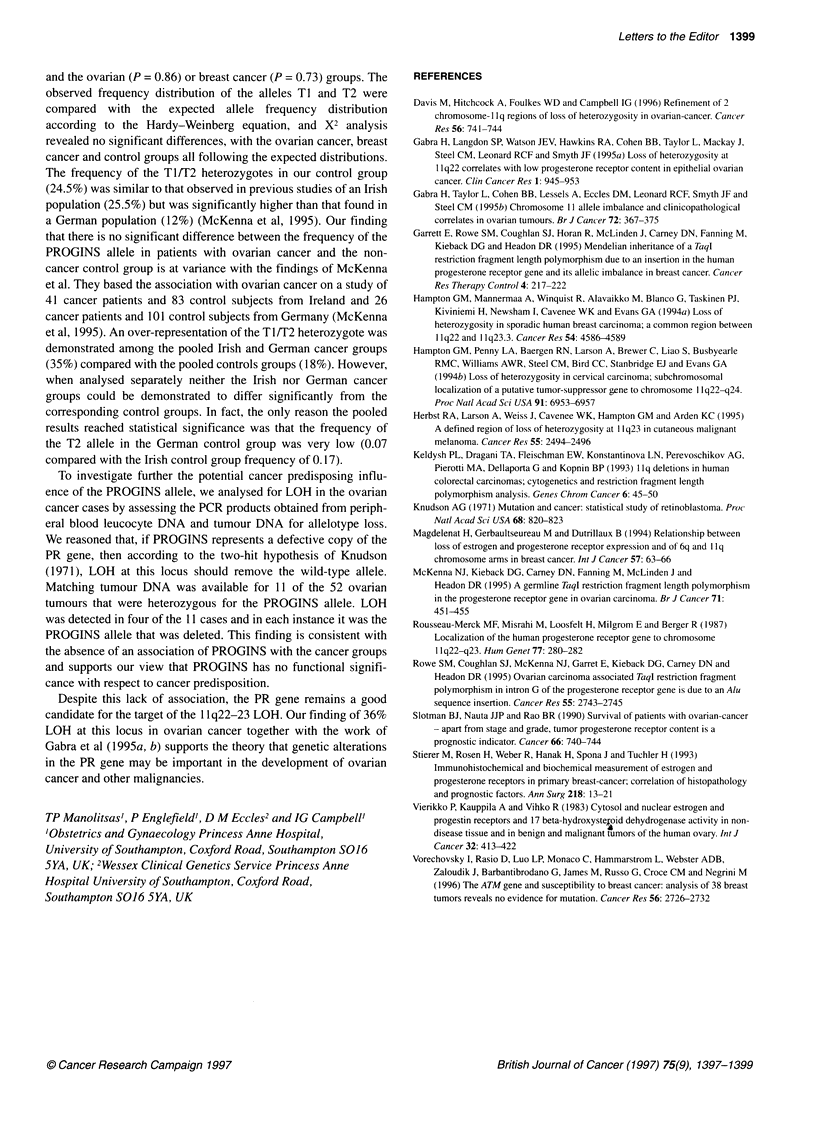# No association of a 306-bp insertion polymorphism in the progesterone receptor gene with ovarian and breast cancer.

**DOI:** 10.1038/bjc.1997.238

**Published:** 1997

**Authors:** T. P. Manolitsas, P. Englefield, D. M. Eccles, I. G. Campbell

## Abstract

**Images:**


### No association of a 306-bp insertion polymorphism in the progesterone receptor gene with ovarian and breast cancer

### Sir

Chromosome region 11q22–23 is a site of frequent loss of heterozygosity (LOH) in breast cancer (Hampton et al, 1994*a*), ovarian cancer (Davis et al, 1996) and other malignancies (Keldysh et al, 1993; Hampton et al, 1994*b*; Herbst et al, 1995), suggesting the presence of an important tumour-suppressor gene(s) (TSG). The most obvious candidate in the region, the gene responsible for ataxia telangiectasia, has recently been ruled out as the target of these deletions in breast cancer (Vorechovsky et al, 1996). The human progesterone receptor (PR) gene is also located in this region (Rousseau-Merck et al, 1987), and there is evidence that it may have tumour-suppressing properties. For example, in ovarian cancer, LOH at the PR locus is associated with reduced PR protein levels (Gabra et al, 1995*a*) and with adverse clinicopathological features (Gabra et al, 1995*b*). Furthermore, there is an association between PR levels and histological subtypes of ovarian cancer (Vierikko et al, 1983). In particular, endometrioid tumours contain elevated PR levels relative to other histological subtypes (Slotman et al, 1990). In breast cancer, similar relationships between PR expression and tumour behaviour (Stierer et al, 1993) and PR expression and 11q LOH (Magdelenat et al, 1994) have been documented. These observations are consistent with a tumour-suppressive role for PR and suggest that it is the target of the 11q22–23 LOH.

In the context of these studies, common polymorphisms of the PR gene that may encode aberrant forms of the PR have been investigated for their association with breast and ovarian cancers. One such polymorphism, designated PROGINS, consists of a 306-bp insertion of the PV/HS-1 *Alu* subfamily in intron G of the PR gene (Rowe et al, 1995). Rowe et al speculated that this insertion might result in expression of an aberrant splice form of PR with altered ligand and hormone-binding properties, since it introduces a consensus splice acceptor site just downstream of a consensus splice donor site. An increased frequency of the PROGINS allele (T2) has been reported in patients with ovarian cancer (McKenna et al, 1995) and breast cancer (Garrett et al, 1995). However, the association with ovarian cancer was based on a total of only 67 cases, 26 from Germany and 41 from Ireland (McKenna et al, 1995). In the breast cancer study, the frequency of the PROGINS allele among 187 cases was increased compared with the 90 controls but did not reach statistical significance (Garrett et al, 1995).

We assessed the frequency of the PROGINS allele in the genomic DNA from 231 sporadic ovarian cancer cases, 292 breast cancer cases and a control group of 220 healthy volunteers in a region of southern England. Previous Polymerase chain reaction (PCR) analyses of the distribution of the T1 and T2 allele used primers that flank regions of 3.0 kb (T2) and 2.7 kb (T1) (Rowe et al, 1995), which were inherently difficult to amplify and resolve on agarose gels. We developed a new set of primers that produce products of 455 bp (T2) and 149 bp (T1), which were amenable to amplification and easily resolved on 1.5% mini agarose gels, as shown in Figure 1. The distribution of the T1 and T2 alleles in the breast cancer, ovarian cancer and non-cancer control groups are shown in Table 1. No significant differences could be demonstrated in the distribution of the alleles between the control group

**Figure 1** Representative agarose gel displaying PCR products of 139 bp (T1) and 445 bp (T2). Homozygotes lacking the 306-bp PROGINS insertion (lanes 1, 2, 3, 5, 6, 7, 8), heterozygotes for the PROGINS insertion (lanes 9 and 10) and a homozygote for the PROGINS insertion (lane 4) are shown

**Table 1** Distribution and frequencies of the PR gene alleles in ovarian cancer, breast cancer and control groups

| Group | Total | $n^a$ | T1/T1 frequency[b] (95% CI)[c] | *n* | Genotype T1/T2 frequency (95% CI) | *n* | T2/T2 frequency (95% CI) | T2 frequency[d] (95% CI) |
|---|---|---|---|---|---|---|---|---|
| Ovarian cancer | 231 | 173 | 74.9 (69–80) | 52 | 22.5 (17–28) | 6 | 2.6 (1–6) | 0.11 (0.09–0.14) |
| Breast cancer | 292 | 229 | 78.4 (74–83) | 61 | 20.9 (16–26) | 2 | 0.7 (0–2) | 0.14 (0.11–0.17) |
| Control group | 220 | 162 | 73.6 (68–79) | 54 | 24.5 (19–30) | 4 | 1.8 (0–5) | 0.14 (0.11–0.17) |

[a]Number of individuals with genotype. [b]Frequency expressed as a percentage of the total. [c]Numbers in parentheses are 95% confidence intervals. [d]Observed allele frequency. Genotypic distributions of breast and ovarian cancer did not differ significantly from control groups (breast cancer, $P = 0.73$; ovarian cancer, $P = 0.86$).

*© Cancer Research Campaign 1997*

and the ovarian ($P = 0.86$) or breast cancer ($P = 0.73$) groups. The observed frequency distribution of the alleles T1 and T2 were compared with the expected allele frequency distribution according to the Hardy–Weinberg equation, and $X^2$ analysis revealed no significant differences, with the ovarian cancer, breast cancer and control groups all following the expected distributions. The frequency of the T1/T2 heterozygotes in our control group (24.5%) was similar to that observed in previous studies of an Irish population (25.5%) but was significantly higher than that found in a German population (12%) (McKenna et al, 1995). Our finding that there is no significant difference between the frequency of the PROGINS allele in patients with ovarian cancer and the non-cancer control group is at variance with the findings of McKenna et al. They based the association with ovarian cancer on a study of 41 cancer patients and 83 control subjects from Ireland and 26 cancer patients and 101 control subjects from Germany (McKenna et al, 1995). An over-representation of the T1/T2 heterozygote was demonstrated among the pooled Irish and German cancer groups (35%) compared with the pooled controls groups (18%). However, when analysed separately neither the Irish nor German cancer groups could be demonstrated to differ significantly from the corresponding control groups. In fact, the only reason the pooled results reached statistical significance was that the frequency of the T2 allele in the German control group was very low (0.07 compared with the Irish control group frequency of 0.17).

To investigate further the potential cancer predisposing influence of the PROGINS allele, we analysed for LOH in the ovarian cancer cases by assessing the PCR products obtained from peripheral blood leucocyte DNA and tumour DNA for allelotype loss. We reasoned that, if PROGINS represents a defective copy of the PR gene, then according to the two-hit hypothesis of Knudson (1971), LOH at this locus should remove the wild-type allele. Matching tumour DNA was available for 11 of the 52 ovarian tumours that were heterozygous for the PROGINS allele. LOH was detected in four of the 11 cases and in each instance it was the PROGINS allele that was deleted. This finding is consistent with the absence of an association of PROGINS with the cancer groups and supports our view that PROGINS has no functional significance with respect to cancer predisposition.

Despite this lack of association, the PR gene remains a good candidate for the target of the 11q22–23 LOH. Our finding of 36% LOH at this locus in ovarian cancer together with the work of Gabra et al (1995*a*, *b*) supports the theory that genetic alterations in the PR gene may be important in the development of ovarian cancer and other malignancies.

*TP Manolitsas[1], P Englefield[1], D M Eccles[2] and IG Campbell[1]*
*[1]Obstetrics and Gynaecology Princess Anne Hospital, University of Southampton, Coxford Road, Southampton SO16 5YA, UK; [2]Wessex Clinical Genetics Service Princess Anne Hospital University of Southampton, Coxford Road, Southampton SO16 5YA, UK*

### REFERENCES

Davis M, Hitchcock A, Foulkes WD and Campbell IG (1996) Refinement of 2 chromosome-11q regions of loss of heterozygosity in ovarian-cancer. *Cancer Res* **56**: 741–744

Gabra H, Langdon SP, Watson JEV, Hawkins RA, Cohen BB, Taylor L, Mackay J, Steel CM, Leonard RCF and Smyth JF (1995*a*) Loss of heterozygosity at 11q22 correlates with low progesterone receptor content in epithelial ovarian cancer. *Clin Cancer Res* **1**: 945–953

Gabra H, Taylor L, Cohen BB, Lessels A, Eccles DM, Leonard RCF, Smyth JF and Steel CM (1995*b*) Chromosome 11 allele imbalance and clinicopathological correlates in ovarian tumours. *Br J Cancer* **72**: 367–375

Garrett E, Rowe SM, Coughlan SJ, Horan R, McLinden J, Carney DN, Fanning M, Kieback DG and Headon DR (1995) Mendelian inheritance of a *Taq*I restriction fragment length polymorphism due to an insertion in the human progesterone receptor gene and its allelic imbalance in breast cancer. *Cancer Res Therapy Control* **4**: 217–222

Hampton GM, Mannermaa A, Winquist R, Alavaikko M, Blanco G, Taskinen PJ, Kiviniemi H, Newsham I, Cavenee WK and Evans GA (1994*a*) Loss of heterozygosity in sporadic human breast carcinoma; a common region between 11q22 and 11q23.3. *Cancer Res* **54**: 4586–4589

Hampton GM, Penny LA, Baergen RN, Larson A, Brewer C, Liao S, Busbyearle RMC, Williams AWR, Steel CM, Bird CC, Stanbridge EJ and Evans GA (1994*b*) Loss of heterozygosity in cervical carcinoma; subchromosomal localization of a putative tumor-suppressor gene to chromosome 11q22–q24. *Proc Natl Acad Sci USA* **91**: 6953–6957

Herbst RA, Larson A, Weiss J, Cavenee WK, Hampton GM and Arden KC (1995) A defined region of loss of heterozygosity at 11q23 in cutaneous malignant melanoma. *Cancer Res* **55**: 2494–2496

Keldysh PL, Dragani TA, Fleischman EW, Konstantinova LN, Perevoschikov AG, Pierotti MA, Dellaporta G and Kopnin BP (1993) 11q deletions in human colorectal carcinomas; cytogenetics and restriction fragment length polymorphism analysis. *Genes Chrom Cancer* **6**: 45–50

Knudson AG (1971) Mutation and cancer: statistical study of retinoblastoma. *Proc Natl Acad Sci USA* **68**: 820–823

Magdelenat H, Gerbaultseureau M and Dutrillaux B (1994) Relationship between loss of estrogen and progesterone receptor expression and of 6q and 11q chromosome arms in breast cancer. *Int J Cancer* **57**: 63–66

McKenna NJ, Kieback DG, Carney DN, Fanning M, McLinden J and Headon DR (1995) A germline *Taq*I restriction fragment length polymorphism in the progesterone receptor gene in ovarian carcinoma. *Br J Cancer* **71**: 451–455

Rousseau-Merck MF, Misrahi M, Loosfelt H, Milgrom E and Berger R (1987) Localization of the human progesterone receptor gene to chromosome 11q22–q23. *Hum Genet* **77**: 280–282

Rowe SM, Coughlan SJ, McKenna NJ, Garret E, Kieback DG, Carney DN and Headon DR (1995) Ovarian carcinoma associated *Taq*I restriction fragment polymorphism in intron G of the progesterone receptor gene is due to an *Alu* sequence insertion. *Cancer Res* **55**: 2743–2745

Slotman BJ, Nauta JJP and Rao BR (1990) Survival of patients with ovarian-cancer – apart from stage and grade, tumor progesterone receptor content is a prognostic indicator. *Cancer* **66**: 740–744

Stierer M, Rosen H, Weber R, Hanak H, Spona J and Tuchler H (1993) Immunohistochemical and biochemical measurement of estrogen and progesterone receptors in primary breast-cancer; correlation of histopathology and prognostic factors. *Ann Surg* **218**: 13–21

Vierikko P, Kauppila A and Vihko R (1983) Cytosol and nuclear estrogen and progestin receptors and 17 beta-hydroxysteroid dehydrogenase activity in non-disease tissue and in benign and malignant tumors of the human ovary. *Int J Cancer* **32**: 413–422

Vorechovsky I, Rasio D, Luo LP, Monaco C, Hammarstrom L, Webster ADB, Zaloudik J, Barbantibrodano G, James M, Russo G, Croce CM and Negrini M (1996) The *ATM* gene and susceptibility to breast cancer: analysis of 38 breast tumors reveals no evidence for mutation. *Cancer Res* **56**: 2726–2732

© Cancer Research Campaign 1997